# Functionality and disease severity in spinocerebellar ataxias

**DOI:** 10.1590/0004-282X-ANP-2020-0580

**Published:** 2022-03-14

**Authors:** Geanison Castro da CRUZ, Marise Bueno ZONTA, Renato Puppi MUNHOZ, Neliana Maria de MELLO, Alex Tiburtino MEIRA, Maria Cristina de Alencar NUNES, Naiara Talita Guimarães ARANHA, Carlos Henrique Ferreira CAMARGO, Francisco Diego Negrão LOPES, Hélio Afonso Ghizoni TEIVE

**Affiliations:** 1 Universidade Federal do Paraná, Hospital de Clínicas, Programa Multiprofissional de Residência Integrada na Atenção Hospitalar, Saúde do Idoso e Adulto, Curitiba PR, Brazil. Universidade Federal do Paraná Hospital de Clínicas Programa Multiprofissional de Residência Integrada na Atenção Hospitalar, Saúde do Idoso e Adulto Curitiba PR Brazil; 2 Universidade Federal do Paraná, Hospital de Clínicas, Departamento de Medicina Interna, Programa de Pós-Graduação em Medicina Interna, Grupo de Doenças Neurológicas, Curitiba PR, Brazil. Universidade Federal do Paraná Hospital de Clínicas Departamento de Medicina Interna Curitiba PR Brazil; 3 University of Toronto, Toronto Western Hospital, Movement Disorders Centre, Toronto ON, Canada. University of Toronto Toronto Western Hospital Movement Disorders Centre Toronto ON Canada; 4 Universidade Federal do Paraná, Hospital de Clínicas, Departamento de Medicina Interna, Serviço de Neurologia, Unidade de Distúrbios do Movimento, Curitiba PR, Brazil. Universidade Federal do Paraná Hospital de Clínicas Departamento de Medicina Interna Curitiba PR Brazil; 5 Universidade Federal do Paraná, Hospital de Clínicas, Residência Médica em Neuropediatria, Curitiba PR, Brazil. Universidade Federal do Paraná Hospital de Clínicas Residência Médica em Neuropediatria Curitiba PR Brazil; 6 Universidade Federal do Paraná, Hospital de Clínicas, Serviço de Estatísticas, Curitiba PR, Brazil. Universidade Federal do Paraná Hospital de Clínicas Serviço de Estatísticas Curitiba PR Brazil

**Keywords:** Spinocerebellar Ataxias, Severity of Illness Index, Postural Balance, Functional Residual Capacity, Ataxias Espinocerebelares, Índice de Gravidade de Doença, Equilíbrio Postural, Capacidade Residual Funcional

## Abstract

**Background:**

Spinocerebellar ataxias (SCAs) are a group of neurodegenerative diseases characterized by deterioration of balance and functionality that tends to follow disease progression. There is no established link between formal clinical markers for severity and functional/balance scores that could guide rehabilitation teams.

**Objective:**

To evaluate the relationship between functional scales and ataxia severity in order to identify cutoff landmarks for functional loss and estimate the mean SARA (Scale for Assessment and Rating of Ataxia) score for the risk ratings for falls on the BBS (Berg Balance Scale).

**Methods:**

Consecutive patients with a molecular diagnosis of SCA (total 89: 31 with SCA2 and 58 with SCA3) were assessed for functionality FIM-ADL (Functional Independence Measure-activities of daily living and Lawton-IADL (instrumental activities of daily living), balance (BBS) and disease severity (SARA).

**Results:**

The main disability cutoff landmarks were that the need for supervision for FIM-ADL starts with 12 points on SARA and the need for supervision for Lawton-IADL starts with 14 points on SARA. The first items to require assistance were “expression” and “shopping”, respectively. At 20 points on SARA, patients were dependent on all FIM and Lawton items. The item with the greatest impact on distinguishing dependents from independents was “means of transport” in Lawton-IADL and the domain “locomotion” in FIM-ADL. The mean SARA score for patients classified as low risk in the BBS was 9.9 points, and it was 17.4 for medium risk and 25.2 for high risk.

**Conclusions:**

Analysis on the correlation between the severity of ataxia and functional scales can form an important guide for understanding the progression of functional dependence among individuals with SCAs.

## RESUMO

**Antecedentes:**

As ataxias espinocerebelares (SCA) são um grupo de doenças neurodegenerativas caracterizadas pela deterioração do equilíbrio e da funcionalidade, que tende a acompanhar a progressão da doença. Não existe uma ligação estabelecida entre os marcadores clínicos formais de gravidade e escores funcionais e de equilíbrio que possam orientar as equipes de reabilitação.

**Objetivo:**

Avaliar a relação entre escalas funcionais e de gravidade da ataxia, buscando identificar pontos de corte para a perda funcional relacionados aos escores de gravidade e aos patamares de Risco de Quedas.

**Métodos:**

Uma amostra consecutiva de 89 pacientes com diagnóstico molecular de SCA (31-SCA2 e 58-SCA3) foram avaliados para funcionalidade MIF-AVDs (Medida de independência funcional-Atividades da vida diária) e Lawton-AIVDs (Atividades instrumentais da vida diária), equilíbrio (EEB-escala de Equilíbrio de Berg), e gravidade da ataxia (SARA-escala para avaliação e graduação de ataxia).

**Resultados:**

Os principais pontos de corte de deficiência foram: com 12 pontos no SARA começa a necessidade de supervisão para MIF-AVDs e com 14 pontos no SARA começa a necessidade de supervisão para Lawton-AIVDs. Os primeiros itens a necessitar de assistência foram “expressão” e “compras”, respectivamente. Com 20 pontos no SARA os pacientes eram dependentes em todos os itens MIF/LAWTON. O item com maior impacto na discriminação entre dependentes e independentes foi “meio de transporte” na Lawton e o domínio “locomoção” na MIF. O escore médio no SARA foi de 9,9 pontos para pacientes classificados com baixo risco na EEB, 17,4 para médio risco e 25,2 para alto risco.

**Conclusões:**

A análise da correlação entre a gravidade da ataxia e as escalas funcionais pode ser um importante guia no entendimento da progressão da dependência funcional em indivíduos com SCA.

## INTRODUCTION

Spinocerebellar ataxias (SCAs) are an expanding group of neurodegenerative diseases characterized by adult onset of symptoms and signs. They result from dysfunction of the cerebellum and its afferent and efferent connections, and also of other central nervous system areas. The area affected and the severity of dysfunction may vary between different types of SCA^1^^,^^2^. Thus, besides cerebellar ataxia, patients may present with oculomotor abnormalities, dysarthria, pyramidal and extrapyramidal signs, pigmentary retinopathy, peripheral neuropathy and cognitive dysfunction, among other manifestations^3^^,^^4^.

At the time of writing of this paper, 48 types of SCAs had been described and classified according to the affected locus^2^. All of them share cerebellar ataxia as the main feature and leading source of disability in most patients affected^1^. As part of the ataxic syndrome, gait instability and impaired coordination are frequently the main subcomponents, thereby leading to overall functional decline and increased dependence^5^.

Assessment of ataxia severity has been used to study long-term disease progression^1^ and to estimate the speed of progression for different types of SCA^1^^,^^6^. Other assessments that quantify patients’ independence for perform basic activities of daily living (ADLs) and instrumental activities of daily living (IADLs), and their balance/risk of falls and quality of life, have also helped multidisciplinary teams to understand the natural history of the disease and its impact on these patients’ daily activities^5^^,^^7^^,^^8^^,^^9^.

The relationship between disease progression and functional loss is relatively well known, but the link between functional scales and ataxia severity has not yet been investigated with the intention of shedding light on which functions and activities are being lost and when this happens over the course of the disease. Better understanding of how functional decline occurs could be used to elaborate and implement clinical practice guidelines and to serve as landmarks for future interventions aimed at preventing and minimizing the impact of the disease.

In this study, we hypothesized that selected formal clinical markers of severity might be directly linked to functional/balance scores. Recognition of the timing and nature of activities that will require some sort of external intervention is the starting point for creation of specific guidelines, including practical and safety strategies for maintaining independence for as long as possible.

The aim of this study was to evaluate the relationship between functional scales and ataxia severity in order to identify cutoff landmarks for functional loss and estimate the mean SARA score for the risk ratings for falls.

## METHODS

We performed prospective data collection between January 2017 and December 2018. This study was approved by the Research Ethics Committee of Hospital de Clínicas, Federal University of Paraná (HC-UFPR). Data were gathered from 89 consecutive patients who were followed at the Ataxia Outpatient Clinic of the Movement Disorder Unit of HC-UFPR.

The sample included subjects aged 18 years or older, irrespective of gender, with a genetically confirmed diagnosis of SCA3 or SCA2, or neurological signs characteristic of ataxia in a family member of a positively tested patient. The choice of SCA3 and SCA2 was based on the fact that these are two of the three most frequent types of SCA in southern Brazil^10^. Moreover, even though the phenotypes of these disorders do not necessarily overlap, they present with similar arrays and severity of motor and nonmotor signs, in addition to having similar ataxia severity progression rates (1.49 and 1.52 for SCA2; 1.56 and 1.60 for SCA3), as described in previous studies^1^^,^^6^. This is in contrast to SCA10, for example, which has a purer phenotype and typically a slower progression rate.

All subjects provided a signed consent statement that was in accordance with the institution’s guidelines. Cases presenting a concomitant clinical disorder that could interfere with the assessments were not included.

### Clinical assessment

Demographic and clinical data were collected during patients’ visits, and were confirmed from the medical records when available. The same examiner assessed all cases. The standardized protocol included obtaining the following demographic data and clinical data: gender, age, age at symptom onset, disease duration, ataxia severity score, family history, details of molecular test, assessment of ataxia severity via the Brazilian version of the Scale for Assessment and Rating (SARA) [consisting of eight items with a final score ranging from 0 (no ataxia) to 40 (most severe)]^11^^,^^12^ and functional scores derived from the Berg Balance Scale (BBS)^13^, Functional Independence Measure (FIM) scale^14^ and Lawton ADLs scale^15^.

The BBS^13^ consists of 14 tasks measuring static and dynamic balance and determining the risk of falls. Scores may range from 0 to 56: individuals with scores between 41 and 56 can walk independently with a low risk of falls; those with scores from 21 to 40 have the ability to walk with assistance and have a medium risk of falls; while those with scores of 20 or lower are wheelchair-bound, with a high risk of falls^13^.

The FIM assesses the amount of assistance required for the individual to carry out ADLs. The maximum score for each of the 18 items is 7, which indicates complete independence, while the minimum score of 1 indicates total dependence. A score of 5 indicates the need for another person to supervise each individual task. The overall score can vary from 18 to 126^14^. In this study, patients were considered independent if they had scores of 6 or 7 for all FIM items.

Lastly, the Lawton IADL scale^15^ is used to assess the ability to live independently and participate in the community (ability to use telephone, shopping, food preparation, housekeeping, laundry, mode of transportation, responsibility for own medications and ability to handle finances). The maximum score on the Lawton scale for IADLs is 21, which indicates complete independence.

### Statistical analysis

The data were tabulated using Microsoft Office Excel 2016^®^ and analyses were performed with the aid of IBM SPSS Statistics v.25.0. (Armonk, NY, USA). Categorical variables were described in terms of frequency and percentage, whereas continuous variables were described in terms of mean and standard deviation. The correlations of FIM, Lawton and Berg scores with the severity of the disease (SARA) was estimated. For this, Spearman’s correlation was used, given that the values ​​on the SARA scale did not present normal distribution. The significance level was taken to be 5%.

The magnitude of the calculated correlations was classified as described by Mukaka^16^. In order to assess the relationship between FIM items and domain classifications, Lawton Scale items and SARA scores, ROC curves were constructed and the AUC and cutoff values ​​were computed. The Kruskal-Wallis test was also used to compare SARA scores between fall risk classification groups (BBS>40, high risk; BBS between 21 and 40, medium risk; BBS<21, high risk). Alpha was taken to be 5% for all analyses performed.

## RESULTS

Among the overall sample of 89 patients evaluated, 45 (50.6%) were male and the mean age was 45.2 (±12.6) years, ranging from 16 to 55 years. There were no significant differences between the two types of SCAs with regard to age (p=0.629), age at symptom onset (p=0.189) or disease duration (p=0.672). Data on molecular characteristics were available for 59 patients (66.3%). Details of the demographic, clinical and molecular characteristics are shown in Table 1. The mean scores for disease severity, functionality and balance for the total sample and for each subdivision according to the risk of falls are shown in Table 2.

**Table 1.** Spinocerebellar ataxia types 2 and 3 -descriptive data.

Variables	SCA2	SCA3	Total	p-value
Patients-	31	58	89	
Sex-Male (%)	19 (61.3)	26 (44.8)	45 (51)	0.139
With genetic testing (%)	17 (54.8)	42 (72.4)	59 (63.3)	0.095
Expansions in altered allele	2240 (2048-2256)	70.5 (67-74)	-	-
Age (years)	44.1±12	45.8±12.9	45.2±12.6	0.629
Age at onset of symptoms (years)	31.7±9.3	34.5±10.2	33.6±9.9	0.189
Disease duration (years)	12.4±7.4	11.2±5.6	11.63±6.3	0.672
Functional scores				
SARA	16 (8.5-24.5)	17 (11-22)	16 (11-23)	0.529
FIM	108 (96.5-117)	105.5 (91.2-113)	106 (95-114)	0.18
Lawton	19 (12.5-21)	17 (14.3-19.8)	18 (13-20)	0.317
Berg	43 (10-51)	34.5 (17-47.5)	38 (15-49)	0.75

Berg: Berg Balance Scale; FIM: Functional Independence Measure (ADL); SARA: Scale for Assessment and Rating of Ataxia.

**Table 2.** Demographic, clinical and functional scores considering the entire sample (n=89) and subdivided according to the risk of falls.

SCA2 and SCA3 (n=89)		Groups according to fall risk			Total
		Low risk	Medium risk	High risk	
AGE (years)		41 (±11.2)	46.2 (±11.3)	50.1 (±13.4)	45.1 (±12.5)
Age at onset of symptoms (years)		32.2 (±9.5)	33.3 (±8.4)	35.4 (±11.3)	33.5 (±9.9)
Disease duration (years)		8.7 (±5.4)	12.8 (±3.9)	14.7 (±6.8)	11.6 (±6.2)
Sara score		9.9 (±5)	17.4 (±3.7)	25.2 (±5.2)	16.5 (±8.3)
FIM total score		114.8 (±8.3)	98.7 (±15.4)	88.3 (±18.7)	102.6 (±18.3)
FIM motor score		82.4 (±6.5)	69.5 (±10.7)	59.6 (±17.3)	72.1 (±15.6)
FIM cognitive score		32.3 (±2.7)	29.2 (±4.8)	28.6 (±3.4)	30.4 (±3.8)
Lawton IADL score		19.8 (±1.6)	19.8 (±1.6)	12.7 (±3.1)	16.7 (±4.1)
Berg score		49.2 (±4.7)	31.1 (±6)	10.4 (±5.6)	32.4 (±18)
Gender	Female	17 (41.4%)	10 (55.5%)	17 (56.6%)	44 (49.4%)
	Male	24 (58.5%)	8 (44.4%)	13 (43.3%)	45 (50.6%)

Berg: Berg Balance Scale; FIM: Functional Independence Measure (Activities Of Daily Living [ADL]); Lawton IADL: Lawton Instrumental Activities of Daily Living Scale; SARA: Scale for the Assessment and Rating of Ataxia.

Considering the entire sample, high negative correlations were identified between SARA scores and FIM (r=-0.730, p<0.001), Lawton (r=-0.808; p<0.001) and BBS scores (r=-0.885, p<0.001).

Table 3 shows the cutoff points of the ROC curve for individual FIM and Lawton items. The first FIM items for which patients needed assistance were “expression” and “dressing lower body”, with loss of independence starting at 12 points in SARA. The last FIM items for which patients lost independence were “toileting” and “bladder management”, with the maximum of 20 points in SARA. It was not possible to verify the influence of the items “social interaction”, “memory” and “understanding” on SARA scale scores, at the 5% significance level.

**Table 3.** Cutoff points for the Receiver Operating Characteristic curve curve for Functional Independence Measure and Lawton items.

Functional independence measure			
FIM dimensions	FIM items	Cutoff SARA	AUC
Self-care	Eating	18.25	0.844
	Grooming	18.5	0.863
	Bathing	17.5	0.883
	Dressing upper body	16.5	0.811
	Dressing lower body	12.75	0.762
	Toileting	20.5	0.773
Sphincter control	Bladder management	18.75	0.762
	Bowel management	16.5	0.656
Transfers	Bed/chair/wheelchair	17.5	0.804
	Toilet	16.5	0.783
	Bath/shower	15.5	0.775
Locomotion	Walking/wheelchair	15.5	0.825
	Stairs	15.5	0.847
Communication	Expression	12.5	0.704
Social interaction	Problem-solving	16.5	0.708
Lawton Instrumental Activities of Daily Living Scale			
Lawton items		Cutoff SARA	AUC
Shopping		14	0.841
Mode of transportation		16.5	0.867
Housekeeping		16.5	0.854
Food preparation		18.5	0.846
Ability to use telephone		20	0.829
Ability to handle finances		18.2	0.815
Responsibility for own medications		16.5	0.844

FIM: Functional Independence Measure (ADL); AUC: area under the curve; SARA: Scale for Assessment and Rating of Ataxia. There was no statistical significance for the items of social interaction, memory and understanding (verbal/nonverbal); these were therefore omitted from the table.

**Figure 1.**
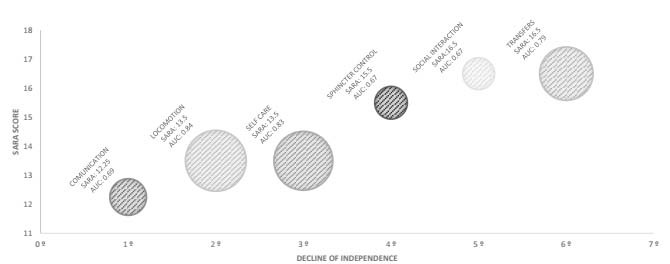
Representation of power of FIM domains to distinguish independence.

AUC: area under the curve; SARA: Scale for Assessment and Rating of Ataxia.

**Figure 2.**
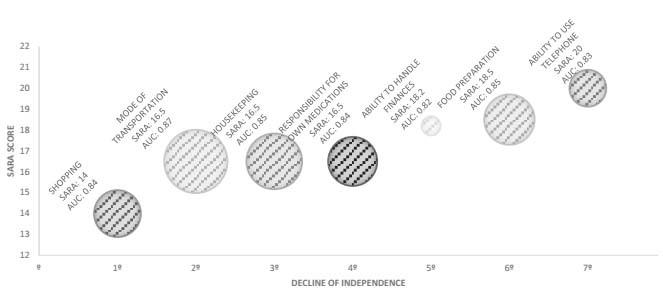
Representation of the power of Lawton scale items to distinguish independence.

AUC: area under the curve; SARA: Scale for Assessment and Rating of Ataxia.

Regarding IADLs, “shopping” was the first activity for which patients with SCA lost their independence, starting from a score of 14 points in SARA. The last skill for which independence was compromised was “ability to use telephone”, with 20 points in SARA. Figure 1 presents the decline in functionality according to FIM domains. The first domain affected was “communication”, which occurred at 12.25 points (AUC: 0.69) in SARA, while the last domain affected was “transfers”, at around 16.5 points. Considering the AUC estimates, it was observed that the domains of “locomotion” (AUC: 0.84), “self-care” (AUC: 0.83) and “transfers” (AUC: 0.79) were the ones with the greatest power to distinguish between dependent and independent patients. In terms of IADLs, the items that showed the greatest power to distinguish between dependent and independent patients were “mode of transportation” (AUC: 0.87), “housekeeping” (AUC: 0.85) and “food preparation” (AUC: 0.85) (Figure 2).

**Figure 3.**
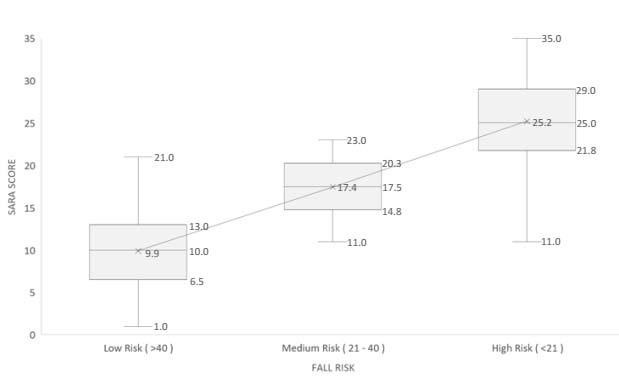
Association between risk of falls (Berg) and severity of ataxia (SARA).

SARA: Scale for Assessment and Rating of Ataxia.

The association between fall risk on the BBS scale and ataxia severity showed that the mean SARA scores were 9.9 points for patients classified as low risk, 17.4 points for those of medium risk and 25.2 points for those of high risk (Figure 3). The fact that there was no overlap between the interquartile ranges that classified the risk of falls demonstrates that there was good segregation between the groups.

## DISCUSSION

Our study confirmed that gradual loss of independence and increased risk of falls are landmarks inherent to the clinical disease progression of patients with SCAs, as observed through the high correlation between increased severity and decreased functional and balance scores. This functional decline is continuous and definitive, progressively compromising patients’ autonomy and quality of life^5^. To date, we have been unable to identify any published data correlating the progression of cerebellar symptoms with loss of independence in specific areas or with the risk of falls.

The present analysis showed that, starting from 12 points on SARA, disability directly related to functional loss already leads patients to require assistance from caregivers, initially in relation to the ability to communicate; and starting from 14 points, for more complex activities. The functional scales that were used in our study provided an overview of how the loss of independence occurs and showed that when 20 points on SARA was reached, patients were already dependent for all the basic and instrumental activities analyzed.

Regarding ADLs, the first item for which SCA patients required assistance from caregivers was “expression”, from the “communication” domain, which was also the first to be affected. The ability to communicate ideas through speech was shown to be one of the leading sources of disability among SCA patients who, due to motor dysfunction in the muscles of the lips, tongue, palatine veil and larynx, present limitations with regard to oral expression^17^. A lack of coordinated muscle contraction for speech articulation is present in ataxias resulting from cerebellar lesions, in which the muscles are hypotonic, thus generating slow and imprecise movements in terms of strength, extension, duration and direction^18^. In addition to the primary disruption to articulation and prosody observed in ataxic dysarthria^19^, many SCA patients also present Schmahmann’s affective-cognitive syndrome^20^, with agrammatism and telegraphic language, but without altered understanding, thus leading to difficulties in oral expression.

Loss of independence regarding expression, as measured by FIM, begins with use of “frequent repetition” to ensure that the essential needs and ideas of everyday life are understood. Needing to repeat frequently and striving to be understood can be tiring and discourage communication, which, in turn, becomes more compromised as the patient becomes isolated. A similar finding was shown by Lirani et al.^21^, in the case of another neurodegenerative disease, Parkinson’s disease. In practical terms, observation of early loss of independence with regard to expression can form a subtle alert for healthcare professionals and caregivers, in that this reflects a need for a detailed assessment of the current overall impact of the disease on ADLs.

Observation of a need for assistance with “eating”, as measured via FIM, in fact refers to a wide range of tasks, such as opening containers, cutting food, passing the butter or serving drinks. It may thus be related to motor coordination difficulties, and not necessarily to dysphagia, and it might be addressed through adaptation using different food types and consistencies. Although dysphagia will settle in, among patients with ataxia, due to mechanisms that also cause dysarthria^17^, the present analysis did not provide any clues as to when these two issues would arise in relation to ataxia severity.

Our study results showed that “dressing lower body” was the next item for which independence became compromised. “Dressing lower body” includes putting on socks, tying shoelaces and wearing pants/trousers. Very early on, when loss of balance becomes significant, patients adapt by sitting down in order to safely perform these activities. This adaptation is considered to represent modified independence. A need for supervision for this task is probably associated with loss of trunk balance, which increases the risk to the patient. This could explain why this specific activity, which is part of the “self-care” domain that was least compromised in our sample, was in isolation the second item to be impacted in terms of independence.

“Bath/shower”, “walking/wheelchair” and “stairs” were the next items for which independence became compromised, at around 15 points on SARA. In a study by Amarante et al.^7^, who evaluated patients with SCA2, the first items to become compromised were, in fact, those related to locomotion (“walking/wheelchair” and “stairs”). Difficulty in walking and climbing stairs limits mobility within different scopes, including social life and access to services in general. The bathroom/shower is one of the house spaces where the greatest numbers of falls take place^22^. Such occurrences should, very early on, serve as a warning for patients with ataxia and their caregivers. For a period, adaptations such as support bars, non-slip mats and use of a bath chair may be sufficient to provide safety, but as the disease progresses, patients will need to be supervised, especially when going into and coming out of the shower cubicle.

The FIM domains of “locomotion”, “self-care” and “transfers” were the ones with the greatest power for distinguishing independence. In a study by Aizawa et al.^5^, for instance, evaluating 44 patients with SCA 1, 2, 3 and 6, the greatest dependence levels were observed in those same domains.

The ability to perform certain tasks, such as “shopping”, “mode of transportation” and “housekeeping” were the first IADLs that patients with SCAs failed to accomplish independently, thus corroborating the study by Santos et al.^8^. These authors evaluated the quality of life in a sample of patients with SCA10, and observed that this variable correlated well with loss of independence to perform these items. In fact, loss of independence regarding activities outside the household environment, especially those that could provide personal satisfaction, leads to progressive isolation, loss of autonomy and loss of quality of life, as also observed in individuals with Parkinson’s disease^23^. In addition to the findings from the study by Santos et al.^8^, we were able to show that these milestones occurred when patients had scores of between 14 and 16 points on SARA. On the other hand, autonomy inside the home, such that patients could take care of their own feeding and the “ability to use telephone” would be markers of dependency with regard to IADLs.

Our data analysis allowed us to present an average value for the SARA score for each level of stratification of the risk of falls in the BBS. These two scales use different approaches to measure the progression of facets of cerebellar signs and symptoms, and combining these parameters provides an integrated assessment of balance and the risk of falls^24^.

Although these assessments of fall risk are important, Van de Warrenburg et al.^25^ warned that even the initial changes to balance control can lead to falls. This issue needs to be brought up among patients and caregivers. Even though higher severity of ataxia is a predictive factor for higher frequency of falls, Fonteyn et al.^26^ found that almost three quarters of their patients reported having had at least one fall in the past 12 months. In most of their patients, falls occurred at early stages of the disease, often within the first two years of the disease. In 10% of their patients, these falls were even the presenting feature^26^. Falls, along with other functional losses, tend to progress inexorably and this also leads to dependency on walking aids (walker/wheelchair). In the present study, this occurred at around 15 points on the SARA scale. Different exercise protocols enable compensation of certain ataxia symptoms, to improve balance and decrease the risk of falls. Thus, rehabilitation constitutes an ally in prolonging the independence of patients with SCA^27^^,^^28^.

To gather a representative sample, the present analysis considered the patients’ disease severity, regardless of the type of SCA and disease duration. Similarly, SCA3 and SCA2 results from monoallelic expansion of a CAG triplet on loci 14q21 and 12q23-24, respectively, were reported^3^^,^^29^^,^^30^. These shared cerebellar ataxia as the main clinical sign, had similar ataxia severity progression rates^1^^,^^6^ and, in the present study, several of their demographic and clinical variables were matched. As such, SARA is an adequate and widely used instrument in both research and routine clinical settings^1^^,^^5^^,^^6^^,^^7^^,^^8^, including for multidisciplinary team assessments and interventions. Our results highlight the importance of keeping track of clinical progression using this scale, considering that, during medical consultations, the rehabilitation team may not always be present. The SARA scores serve as a parameter that can be correlated with more functional outcomes.

Grouping together two different SCAs in a single study might be seen as a limitation, but in fact it was a strategy for optimizing the statistical power of our study. It needs to be considered that the sequences within which different items in the SARA are affected may differ. For example, there is earlier involvement of the lower limbs in SCA3^31^, which could reflect specific types of impaired activities.

Future studies with larger samples that provide adequate statistical power to analyze different SCA subtypes may show whether functional differences exist among these disorders. Hopefully, such studies will also show their correlations and score thresholds on SARA. We believe that the greatest contribution of the present analysis was that it enabled a more functional and practical view of patients, using a parameter for severity that was derived from a reliable and easy-to-apply instrument such as SARA.
